# Association of health-related quality of life with urinary tract infection among kidney stone formers

**DOI:** 10.1007/s00240-024-01601-3

**Published:** 2024-07-03

**Authors:** Mingrui Wang, Chin-Hui Lai, Jiaxiang Ji, Haopu Hu, Runfeng Ni, Jun Liu, Luping Yu, Hao Hu

**Affiliations:** 1 Department of Urology, Peking University People’s Hospital, Beijing, China; 2 The institute of applied lithotripsy technology, Peking University, Beijing, China

**Keywords:** Urolithiasis, Urinary tract infection, Kidney stone disease, Quality of life

## Abstract

Kidney stones and infections significantly affect patients’ health-related quality of life (HRQOL); however, the relationship between urinary tract infections (UTIs) and HRQOL in patients with kidney stones remains unclear. This study aimed to investigate the relationship using the validated Chinese version of the Wisconsin Stone Quality of Life questionnaire (C-WISQOL). We prospectively recruited 307 patients with kidney stones to complete the C-WISQOL before and after stone removal. The participants were diagnosed with UTI based on the presence of pyuria or bacteriuria with or without clinical symptoms. The psychometric properties of the C-WISQOL were statistically analyzed. Multivariate linear regression was used to predict the risk factors for impaired HRQOL in patients with stones and UTIs. The questionnaire is a reliable and robust tool for evaluating HRQOL in Chinese-speaking patients with urolithiasis. The UTI and kidney stone co-occurrence was significantly associated with female sex, diabetes mellitus, more previous stone events, higher antibiotic usage, positive stone- or UTI-related symptoms, and postoperative residual stones. The preoperative C-WISQOL scores and improvement in the HRQOL after stone removal in patients clinically diagnosed with UTI were significantly inferior to those in patients without UTI. The regression analyses showed that worse HRQOL was predicted by more previous stone events and positive stone- or UTI-related symptoms. In contrast, the presence of diabetes mellitus and postoperative residual stone fragments predicted a lower improvement in the HRQOL. These findings underscore UTI’s harmful impact on perioperative HRQOL in patients with kidney stones and could help strategies benefit those patients.

## Introduction

The global prevalence of urolithiasis has been rising rapidly in recent decades, attracting increased attention to the health-related quality of life (HRQOL) of the affected individuals. Evidence indicates that patients with urinary stones are associated with poor HRQOL versus the national average population [1, 2]. However, the risk factors contributing to HRQOL deterioration are mixed and complicated. Urinary stone disease and the related complications and concurrent comorbidities significantly affect individuals’ physical and mental well-being and their daily lives and professional activities [3, 4]. Medications and surgical interventions have shown promise in significantly improving patients’ HRQOL [5].

Urinary tract infections (UTIs) are associated with urolithiasis. Although urinary pathogens are implicated in the formation of stones in approximately 15% of cases, infections co-occur in up to 50% of patients with urinary stones [6, 7]. A systematic review concluded that patients with UTI tend to have significantly lower HRQOL scores compared with those without UTI [8]. Jeffrey et al. reported that uncomplicated UTIs were associated with impaired activities, decreased productivity, and a reduced QOL, as assessed by the Short Form 36 Health Survey (SF-36) [9]. Symptomatic or recurrent UTIs increase patient discomfort and stress or cause the relapse of concomitant stones. However, few studies have evaluated the relationship between UTI and the HRQOL in patients with urological stones. Despite the surgical benefits for improving HRQOL among patients with kidney stones are clear, the impact of UTI on the HRQOL improvement caused by stone removal surgery is still unknown.

The Wisconsin Stone Quality of Life (WISQOL) questionnaire is a disease-specific tool for measuring the HRQOL in individuals with urolithiasis [10, 11]. It consists of 28 questions organized into four subdomains: social impact, emotional impact, stone-related symptom impact, and vitality impact. Total scores on the WISQOL range from 28 to 140 points, with higher scores indicating better HRQOL [12–14]. However, the Chinese version of the WISQOL (C-WISQOL) is has been rarely evaluated. In this study, we used a standard cross-cultural translation process to verify the reliability and validity of C-WISQOL, aiming to investigate the relationship between UTI and the HRQOL in patients with urolithiasis, based on data from a large cohort of patients with urinary stones. We also explored the risk factors contributing to the impaired HRQOL of patients with stones and UTIs. The study’s findings could provide insight into developing new strategies for improving the management and treatment of urolithiasis and UTI.

## Patients and methods

### Study design and population

Following the initial authorization for the translation and verification of the WISQOL, we adopted the standard cross-cultural adaptation of the HRQOL measures to develop the C-WISQOL [15]. In the pilot study, ten patients with kidney stones completed the C-WISQOL questionnaire, confirming its readability and comprehensibility. Participants aged 18–80, diagnosed with kidney stones, and possessing sufficient proficiency in the Chinese language were recruited between July 2022 and June 2023. Patients with ureteral stents, malignant tumors, urosepsis, or incomplete questionnaires were excluded. All patients provided written informed consent for anonymized evaluation and publication of their data. The study was approved by the Ethics Committee of Peking University People’s Hospital (2020PHB177-01).

### Data collection

Patient demographic and perioperative characteristics, including sex, age, comorbidities, stone history, history of preoperative pyelonephritis, antibiotics and analgesics usage, symptoms, surgical methods used, and presence of postoperative residual stones, were recorded. During the first visit to the urological outpatient clinic or emergency room, patients completed the C-WISQOL-1 and SF-36 v2. Two weeks later, the C-WISQOL-2 was completed before surgery. Patients were then categorized into two groups: a UTI group (patients with UTIs) and a no-UTI group (patients without UTIs), according to preoperative diagnostic criteria for UTI, which include pyuria (> 10 white blood cells/mm^3^ per HPF) or bacteriuria (≥ 10^5^ CFU/mL), with or without accompanying clinical symptoms. Finally, the C-WISQOL-3 was filled out after kidney stone and ureteral stent removal surgeries.

### Statistical analyses

All statistical analyses were performed using SPSS version 22. Descriptive statistics included the frequencies and proportions of categorical variables, and means with standard deviations were reported for the measurement variables. Cronbach’s alpha and Spearman’s rho were used to verify the internal consistency and associations of the C-WISQOL, respectively. Spearman’s rho was used to analyze the association between the C-WISQOL and SF-36 v2 to evaluate convergent validity. The Wilcoxon signed-rank test was used to assess the test-retest reliability and sensitivity of treatment changes. Cronbach’s alpha values were interpreted as follows: unacceptable (< 0.50), poor (0.50–0.60), questionable (0.61–0.70), acceptable (0.71–80), good (0.81–0.90), and excellent (> 0.90). The Spearman’s rho values were interpreted as follows: poor (≤ 0.20), fair (0.21–0.40), moderate (0.41–0.60), good (0.61–0.80), and excellent (> 0.80). The Student’s t-tests or χ^2^ tests were used to assess differences in demographic characteristics and the C-WISQOL scores between the two groups. Multivariate linear regression was used to predict the risk factors contributing to impaired HRQOL in patients with stones and UTIs. Statistical significance was set at *p* < 0.05.

## Results

Of the 337 patients with kidney stones initially enrolled in this study, ten had indwelling ureteral stents upon their first visit, five developed urosepsis after stone surgeries, and five had incomplete survey data. Therefore, 307 patients were included in the final analyses. Their mean age was 51.9 (range 18–80) years, and the average body mass index was 25.4 kg/m^2^. Among them, 134 (43.6%) were male, and 173 (56.4%) were female.

The Cronbach’s alpha for the total C-WISQOL questionnaire was 0.968, indicating excellent internal consistency. Subdomains scores were as follows: 0.898, 0.892, 0.860, and 0.880 for the social, emotional, stone-related, and vitality impact, respectively. The inter-domain Spearman’s rho within the C-WISQOL ranged from 0.703 to 0.846 (Table 1), and all domain scores were strongly correlated with the total score (Spearman’s rho of 0.904, 0.900, 0.875, and 0.875, respectively). All correlations between the corresponding domains in the C-WISQOL and SF-36 v2 questionnaires evaluated using Spearman’s rho ranged from 0.684 to 0.901, indicating good convergent validity. However, only a moderate correlation was observed between the total scores of the two questionnaires (Spearman’s rho = 0.564), specifically assessing stone-related HRQOL (Table 2). Comparison of the total score and each subdomain score between the two C-WISQOL administrations, two weeks apart, showed similar results (all *p* > 0.05), indicating reliable test-retest consistency. In addition, the C-WISQOL was sensitive to change, as significant differences were observed in the total and subdomain scores between the preoperative and postoperative assessments (*p* < 0.05) (Table 3).

**Table 1:** Interdomain and domain-total associations of the Chinese version of the Wisconsin Stone Quality of Life-1

Domain	D1	D2	D3	D4
Social impact D1	1			
Emotional impact D2	0.799	1		
Stone-related impact D3	0.731	0.703	1	
Vitality impact D4	0.804	0.846	0.720	1
Total score	0.904	0.900	0.875	0.875

C-WISQOL-1, Chinese version of the Wisconsin Stone Quality of Life-1

**Table 2:** Convergent validity of the Chinese version of the Wisconsin Stone Quality of Life-1 and short form 36 Health Survey version 2

C-WISQOL-1	SF-36 v2	Spearman’s rho
Social impact D1	Social functioning: 60.0 ± 13.4	0.724
Emotional impact D2	Mental health: 59.3 ± 12.4	0.800
Stone-related impact D3	General health: 59.4 ± 12.4	0.684
Vitality impact D4	Energy vitality: 78.9 ± 16.5	0.901
Total score	Total score: 108.7 ± 13.9	0.564

C-WISQOL-1, Chinese version of the Wisconsin Stone Quality of Life-1; SF-36 v2, Short Form 36 Health Survey version 2

**Table 3:** Test-retest reliability and sensitivity to change in the Chinese version of the Wisconsin Stone Quality of Life scores

Domain	C-WISQOL-1	C-WISQOL-2	p1	C-WISQOL-3	p2
Social impact D1	27.2 ± 4.2	27.3 ± 4.4	**0.845**	33.1 ± 3.3	**<0.001**
Emotional impact D2	23.8 ± 3.7	23.9 ± 3.5	**0.791**	29.0 ± 3.0	**<0.001**
Stone-related impact D3	27.0 ± 3.6	27.3 ± 4.1	**0.494**	33.8 ± 4.1	**<0.001**
Vitality impact D4	10.1 ± 1.9	10.2 ± 1.7	**0.901**	12.5 ± 1.8	**<0.001**
Total score	94.9 ± 13.7	95.5 ± 14.3	**0.709**	116.7 ± 12.3	**<0.001**

p1: C-WISQOL-2 vs. C-WISQOL-1; p2: C-WISQOL-3 vs. C-WISQOL-2; C-WISQOL, Chinese version of the Wisconsin Stone Quality of Life

A total of 131 (30.0%) patients with kidney stones were simultaneously diagnosed with UTI (Table 4). The percentage of women and individuals with diabetes mellitus significantly differed between the UTI and no-UTI groups (63.4% vs. 51.1%, *p* = 0.033; 29.0% vs. 17.0%, *p* = 0.013, respectively). Although the duration of previous stone disease was similar (*p* = 0.204), patients with UTIs experienced more stone events (3.9 vs. 2.0, *p* < 0.001) and were prescribed more antibiotics (77.1% vs. 60.2%, *p* = 0.002) compared with those without UTIs. The study population demonstrated a significant difference in stone- or UTI-related symptoms between the groups, with 80.2% of patients with UTI-related symptoms compared with 52.8% of patients without UTIs (*p* < 0.001). The percentage of previous analgesics usage also significantly differed between UTI group and No-UTI group (75.6% vs. 48.3%, *p* < 0.001). Although the two groups underwent similar stone removal surgeries (*p* = 0.133), a higher proportion of patients with UTIs had postoperative residual stones compared with patients without UTIs (40.5% vs. 24.4%, *p* = 0.003). No significant difference was observed in the stone composition between the UTI and no-UTI groups (*p* = 0.241).

**Table 4:** Demographic and clinical characteristics of the patients with kidney stones

Parameter	UTI group	No-UTI group	*p*-value
Cases	131	176	NA
Sex Male Female	48 83	86 90	**0.033**
Age (y)	52.2 ± 13.1	51.7 ± 13.8	0.718
BMI (kg/m^2^)	24.8 ± 3.7	25.4 ± 3.3	0.175
Diabetes mellitus Yes No	38 93	30 146	**0.013**
Stone disease duration	12 (0.2–420)	8.5 (0.2–400)	0.204
Stone events number	3.9 (1–15)	2.0 (1–8)	**< 0.001**
History of pyelonephritis Yes No	13 118	10 166	0.163
Prior antibiotics uses Yes No	101 30	106 70	**0.002**
Prior analgesics uses Yes No	99 32	85 91	**< 0.001**
Symptoms Yes No	105 26	93 83	**< 0.001**
Stone removal procedure PCNL RIRS	58 73	63 113	0.133
Stone composition Infectious stone Noninfectious stone	26 105	26 150	0.241
Postoperative residual stones Yes No	53 78	43 133	**0.003**

UTI, urinary tract infection; BMI, body mass index; PCNL, percutaneous nephrolithotomy; RIRS, retrograde intra-renal surgery. Bold text indicates statistical significance

Among patients with UTIs, a significant decline was observed in the preoperative total C-WISQOL score (92.5 vs. 96.6, *p* = 0.01) as well as a decline across each of its four subdomains (*p* < 0.05), especially the vitality domain score (*p* < 0.005) (Table 5). In addition, the postoperative total C-WISQOL score and each of its subdomain scores in the UTI group were significantly lower than those in the no-UTI group (all *p* < 0.005). Following the intervention, significant differences were observed between patients with and without UTI in terms of the total C-WISQOL scores (112.9 vs. 92.5, *p* = 0.001 for the UTI group; 118.6 vs. 96.6, *p* < 0.001 for the no-UTI group). This finding was consistent and maintained significance across all four subdomains of the C-WISQOL (*p* < 0.005). Regarding the changes in the C-WISQOL, only the changes in the total score and social impact domain score were significantly different between the UTI and no-UTI groups (20.4 vs. 22.0, *p* = 0.040; 5.4 vs. 6.0, *p* = 0.042, respectively). No significant differences were observed in the changes in the C-WISQOL in terms of social impact (*p* = 0.360), emotional impact (*p* = 0.121), or stone-related symptom impact subdomains (*p* = 0.954).

**Table 5:** The Chinese version of the Wisconsin Stone Quality of Life scores of the patients with kidney stones

Parameter	UTI group	No-UTI group	*p*-value
C-WISQOL-1 D1 D2 D3 D4 Total scores	26.5 ± 4.2 23.2 ± 3.8 26.4 ± 3.8 9.8 ± 1.9 92.5 ± 14.2	27.7 ± 4.1 24.1 ± 3.6 27.5 ± 3.3 10.4 ± 1.8 96.6 ± 13.2	**0.017** **0.034** **0.005** **0.004** **0.010**
C-WISQOL-3 D1 D2 D3 D4 Total scores	31.9 ± 3.4 28.2 ± 3.1 32.7 ± 4.3 12.1 ± 1.9 112.9 ± 12.8	33.6 ± 3.2 29.4 ± 2.9 34.4 ± 4 12.7 ± 1.8 118.6 ± 12	**< 0.001** **0.001** **0.001** **0.004** **< 0.001**
Change of C-WISQOL D1 D2 D3 D4 Total scores	5.4 ± 2.7 5 ± 2.2 6.3 ± 2.9 2.3 ± 0.9 20.4 ± 6.9	6 ± 2.4 5.2 ± 2.2 6.8 ± 2.8 2.3 ± 0.9 22 ± 6.4	**0.042** 0.360 0.121 0.954 **0.040**

UTI, urinary tract infection; C-WISQOL, Chinese version of the Wisconsin Stone Quality of Life. Bold text indicates statistical significance

Among patients with stones and UTIs, multivariate linear regression analysis indicated that the preoperative C-WISQOL score was significantly associated with the number of previous stone events and the presence of stone or UTI symptoms (*p* < 0.001 and *p* = 0.004, respectively) (Table 6). Those with stones or related symptoms exhibited lower preoperative HRQOL, with a more pronounced decline as the number of stone events increased. The presence of diabetes mellitus and postoperative residual stones were identified as significant factors influencing the changes in the C-WISQOL scores after the interventions compared with those before them (*p* = 0.047 and *p* < 0.001, respectively). Patients without diabetes mellitus who achieved a stone-free status following the surgical procedures showed a recovery in the total HRQOL during follow-up.

**Table 6:** Multivariate linear regression to predict the risk factors for impaired health-related quality of life in stone formers with urinary tract infections

Coefficient	C-WISQOL-1		Change in C-WISQOL	
	Beta	*p* value	Beta	*p*-value
Age	-0.068	0.375	0.064	0.439
Sex	-0.070	0.365	-0.042	0.609
Diabetes mellitus	-0.050	0.514	**-0.168**	**0.047**
Stone events number	**-0.389**	**< 0.001**	0.031	0.704
Symptoms	**-0.235**	**0.004**	0.066	0.424
Postoperative residual stones	NA	NA	**-0.406**	**< 0.001**
R^2^	0.260		0.157	
Adjusted R^2^	0.248		0.144	
F	22.439		11.950	
Significance of F	< 0.001		< 0.001	

C-WISQOL, Chinese version of the Wisconsin Stone Quality of Life. Bold text indicates statistical significance

## Discussion

The presence of urinary stones significantly contributes to a reduced HRQOL, with several factors exacerbating this decline [1–4]. We developed a Chinese version of the WISQOL according to the standard protocol for cross-cultural adaptation of quality of life measurement, which is a valid tool for evaluating patient HRQOL in Chinese-speaking patients with kidney stones. Among the 307 patients with kidney stones who completed the C-WISQOL, clinically diagnosed UTI was strongly associated with impaired HRQOL and relatively less improvement after surgical treatment.

Chinese is the most widely spoken language worldwide; therefore, this comprehensive study provides a reliable and easy-to-read instrument for assessing disease-specific HRQOL among Chinese speakers. The C-WISQOL exhibited excellent internal consistency, especially in the total score of the questionnaire (Cronbach’s alpha > 0.95), which was comparable to the results of the original WISQOL and its validated versions in other languages [11, 16, 17]. Consistent with previous studies, the interdomain reliability analysis indicated a good correlation among the subdomain scores and an excellent correlation between the subdomain and total scores [16, 17]. In addition, the C-WISQOL presented reliable test-retest results with a 2-week interval for each subdomain and the entire questionnaire. Following the kidney stone removal surgery, the questionnaire scores demonstrated sensitivity to changes, effectively distinguishing between patients with and without kidney stones. The convergent validity of the C-WISQOL was confirmed through its strong correlation with the corresponding subdomains of the SF-36 v2. However, the relatively weaker correlation between the total scores of the two questionnaires indicated the C-WISQOL’s superior sensitivity as a disease-specific HRQOL measurement for kidney stone condition, compared to the general SF-36 v2. These findings suggest that the C-WISQOL is a reliable tool for evaluating the HRQOL in patients with urinary stones, offering a potentially valuable assessment of QOL for patients with urinary stones in China.

UTIs rank among the most common bacterial infections acquired in both community and hospital settings. Infection is implicated as the cause of stones in approximately 15% of patients with stones, whereas the simultaneous occurrence of infection and stones has been reported in up to 50% of patients [6, 7], which is consistent with the findings of our study (30%). In this analysis, female patients with stones and those with diabetes mellitus were significantly associated with confirmed UTIs simultaneously. These findings are consistent with those of previous studies suggesting that female sex, glucose metabolic abnormalities, and positive urine culture are significant independent predictors of increased UTI risk after ureteroscopy for kidney stone diseases [18, 19]. The present study revealed that the majority of patients with kidney stones and concurrent UTIs experienced disease-related symptoms, further supporting the hypothesis that the combination of kidney stones and infection could have a negative impact on a patient’s physical health. The finding of a more pronounced association between the number of previous stone events or postoperative residual stones and infections among patients with kidney stones was consistent with several studies [20, 21], which supported that recurrent lifetime stone events were significant risk factors contributing to complicated UTIs.

These results are broadly consistent with those of previous studies that examined different demographic factors or comorbidities affecting the QOL of individuals with kidney stones [12–14]. Qualitatively, the results among patients with kidney stones who completed the C-WISQOL were consistent with previous findings indicating that stone formers experience worse HRQOL in the presence of stress, frequent stone occurrence, longer duration of stone disease, and increased travel distance for treatment [3, 4, 14, 22]. The current study observed a significant difference in the C-WISQOL scores between kidney stone formers with and without UTI, especially in the stone-related symptom impact and vitality impact subdomains. A notable distinguishing feature of the current study was its evaluation of the postoperative HRQOL and change in the HRQOL, suggesting that UTI not only impairs the QOL of patients with kidney stones but also has an adverse effect on the improvement of QOL after stone removal procedures. Importantly, the presence of UTI significantly impacted the improvement of social subdomain, which might because that patient’s postoperative social activity was still restricted owing to persistent and recurrent urine disturbance. However, the change in terms of emotion, stone-related symptom and vitality caused by surgical interventions was not associated with concurrent UTI.

Due to the chronic and sporadic progression of kidney stone disease, predicting how patients experience their illness and what impacts their QOL is challenging. Our study identified the number of previous stone events and current stone- or UTI-related symptoms as significant independent risk factors for poor HRQOL in patients with kidney stones and UTIs. Considering that Kristina et al. found a worse HRQOL among those with symptomatic stones [10, 23], we speculated that the decreased HRQOL in patients with kidney stones and concurrent infections was partially due to the impact of stone- or UTI-related symptoms. In addition, the higher the number of stone events, the more likely the patients were to experience lower HRQOL, a finding supported by Tapiero et al.’s study indicating that more than five lifetime stone events were associated with a lower QOL [14].

Although stone removal surgeries significantly improve patients’ QOL, individuals with simultaneous UTI experience fewer changes in the HRQOL improvement. The presence of diabetes mellitus and postoperative residual fragments, both significant independent predictors, may explain this disparity in the HRQOL outcomes. In a study examining the QOL of patients with kidney stones who also have metabolic syndromes, diabetes mellitus, and body mass index of > 30 kg/m^2^ were associated with worse HRQOL. This association may be attributed to diabetes mellitus being linked with an increased risk of nephrolithiasis, negatively impacting the stone-specific QOL [24]. In contrast to our findings, Streeper et al. reported that a stone-free status after surgical intervention was not significantly associated with a better HRQOL [25]. However, the present study illustrated that patients with kidney stones and simultaneous UTIs experienced more postoperative residual stones than those without UTIs, which may help answer the question of whether the decreased HRQOL was possibly caused by the postoperative residual fragments. Therefore, we posit that the presence of diabetes mellitus and postoperative residual stones could compromise the improvement in the HRQOL following stone removal surgeries in patients with stones and UTIs. Overall, more evidence is needed using consistent measures of stone-related QOL throughout the entire duration of the stone event to understand better the clinical importance of the risk factors for elevating patient HRQOL during the stone formation event.

This study has limitations. First, the generalizability of the results to other regions and institutions may be limited due to the single-center nature of the study. Second, multivariate regression analysis was not conducted for each domain of the C-WISQOL. Therefore, it was not possible to measure the specific impact of UTI on the HRQOL in patients with kidney stones. However, we have demonstrated that the C-WISQOL had good interdomain and domain-total associations, which may mitigate these concerns. Third, due to financial constraints, computerized tomography imaging, known for its sensitivity in detecting postoperative residual stone fragments, was not included as a secondary outcome. Instead, alternative modalities, such as kidney-ureter-bladder radiography and abdominal ultrasound, were used, similar to many other studies. Despite these limitations, the study’s major strengths lie in the robust methods used to validate the C-WISQOL questionnaire and identify the risk factors for a reduced HRQOL and its lower improvement in patients with kidney stones and UTIs.

## Conclusions

The C-WISQOL questionnaire is a reliable tool for evaluating the HRQOL in Chinese-speaking patients with urolithiasis. Among patients with kidney stones who completed the C-WISQOL, clinically diagnosed UTI was significantly associated with a poorer HRQOL, predicted by a history of more previous stone events and positive stone- or UTI-related symptoms. The presence of diabetes mellitus and postoperative residual stone fragments predicted a lower improvement in the HRQOL. Based on the findings from this study, novel approaches could be developed to benefit the treatment of kidney stones and UTIs.

## Data Availability

Data is provided within the manuscript or supplementary information files.
